# Asymmetric and Symmetric Protein Arginine Dimethylation: Concept and Postprandial Effects of High-Fat Protein Meals in Healthy Overweight Men

**DOI:** 10.3390/nu11071463

**Published:** 2019-06-27

**Authors:** Alexander Bollenbach, Jean-François Huneau, François Mariotti, Dimitrios Tsikas

**Affiliations:** 1 Institute of Toxicology, Core Unit Proteomics, Hannover Medical School, 30623 Hannover, Germany; 2 UMR PNCA, AgroParisTech, INRA, Université Paris-Saclay, 75005 Paris, France

**Keywords:** ADMA, arginine, SDMA, DMA, PRMT

## Abstract

Asymmetric and symmetric dimethylarginine (ADMA and SDMA, respectively) are risk factors for the cardiovascular and renal systems. There is a paucity of data in humans regarding variations of protein L-arginine (Arg) methylation leading to ADMA and SDMA. In this study, we introduced and used Arg dimethylation indices based on the creatinine-corrected urinary excretion of SDMA and ADMA, and its major metabolite dimethylamine (DMA). The main objective of the present study was to assess whether, and to which extent, a high-fat protein meal (HFM), a classical allostatic load eliciting various adverse effects, may contribute to Arg dimethylation in proteins in humans. Reliable gas chromatography–mass spectrometry methods were used to measure the concentration of ADMA, DMA, SDMA, and creatinine in spot urine samples collected before (0 h), and after (2, 4, 6 h) three HFM sessions in 10 healthy overweight individuals. At baseline, urinary ADMA, DMA, and SDMA excretion correlated positively with circulating TNF-α and IL-6. Arg dimethylation indices did not change postprandially. Our study shows that three HFMs do not contribute to Arg dimethylation in proteins. The proposed indices should be useful to determine extent and status of the whole-body Arg dimethylation in proteins in humans under various conditions.

## 1. Introduction

**Figure 1:**
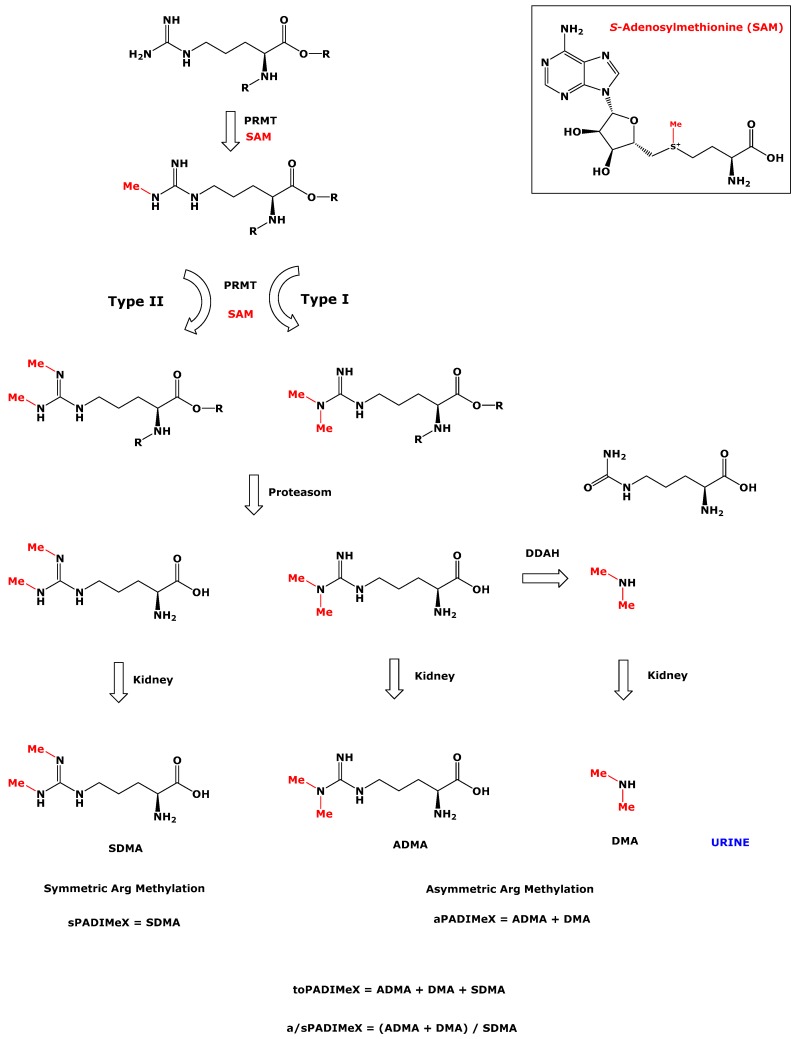
Simplified schematic of the asymmetric and symmetric methylation (ADMA and SDMA respectively) of arginine residues in proteins, their proteolysis to free ADMA and SDMA, metabolism of ADMA by dimethylarginine dimethylaminohydrolase (DDAH) to dimethylamine (DMA), and their excretion in the urine. Proposal of the protein arginine dimethylation index (PADiMeX).

L-Arginine (Arg) is a nutritionally semi-essential proteinogenic amino acid. It is involved in many pathways and numerous physiological processes [1,2]. Arg is the substrate of all known nitric oxide synthase (NOS; EC 1.14.13.39) isoforms, which are present in virtually all cell types and oxidize the guanidine (*N*^G^) imine group of free Arg to nitric oxide (NO) via an *N*^G^-hydroxy-L-arginine intermediate. NO is one of the most potent endogenous vasodilators and inhibitors of platelet aggregation, and has many other biological functions [3]. The NOS-catalyzed conversion of Arg to NO and L-citrulline (Cit) is inhibited by three endogenous Arg derivates: L-*N*^G^-monomethylarginine (MMA), L-*N*^G^,*N*^G^-dimethylarginine (asymmetric dimethylarginine, ADMA), and L-*N*^G^,*N’*^G^-dimethylarginine (symmetric dimethylarginine, SDMA) [4,5] (Figure 1). MMA, ADMA, and SDMA inhibit the activity of the three NOS isoforms by distinctly different mechanisms and inhibitory potency [6,7]. Like their parent molecule Arg, MMA, ADMA, and SDMA exist in two forms: as residues of certain proteins, and as free acids produced by regular proteolysis of those *N*^G^-methylated proteins. The free guanidine group of Arg moieties in proteins undergo posttranslational methylation, which is catalyzed by the family of the protein arginine methyltransferases (PRMT; EC 2.1.1.125) [8,9,10]; the methyl group for this reaction is provided by the universal cofactor *S*-adenosylmethionine (SAM) (Figure 1).

High circulating ADMA and SDMA concentrations are considered risk factors in the renal and cardiovascular systems [11,12,13,14,15,16]. In these systems, ADMA is thought to exert its detrimental effects by inhibiting NOS activity in the endothelium. Yet, there is an increasing indication that free and/or proteinic ADMA and SDMA exert NO-independent biological effects that have not yet been fully elucidated [6,7]. As an example, mass proteomic studies identified mono- and dimethylated Arg residues in the cardiac sodium channel, suggesting a potential role of proteinic Arg methylation in the regulation of the cardiac voltage-gated Na^+^ channel, presumably via mutual Arg methylation–phosphorylation crosstalk [17,18,19]. It has also been discussed that methylation of arginine residues in proteins are essential for proper regeneration of skeletal muscles, presumably by regulating muscle stem cell function [9].

A major fraction (about 90%) of endogenously produced ADMA is hydrolyzed to dimethylamine (DMA) by dimethylarginine dimethylaminohydrolase (DDAH; EC 3.5.3.18); only a minor fraction of about 10% of the daily produced ADMA is excreted, unchanged, in the urine [4,20]. Unlike MMA and ADMA, SDMA is not hydrolyzed by DDAH and is excreted almost unchanged in the urine [21]. The urinary concentrations of ADMA, DMA, and SDMA can be considered markers of whole-body asymmetric (ADMA + DMA), symmetric (SDMA), and total (ADMA + DMA + SDMA) dimethylation of Arg residues in proteins. Therefore, this could be a practical way to assess whole-body Arg dimethylation, and its variations in vivo. Previously, we and others have used Arg methylation indices for symmetric [22] and asymmetric Arg dimethylation, including the DMA/ADMA molar ratio in urine of healthy and diseased children and adults [23,24]. In the present work, we further build on the rationale of indices for assessing protein Arg dimethylation by defining and using the following terms: aPADiMeX for asymmetric dimethylation; sPADiMeX for symmetric dimethylation; toPADiMeX (ADMA + DMA + SDMA) for total Arg dimethylation; and a/sPADiMeX for the molar ratio of asymmetric-to-symmetric Arg dimethylation, i.e., (ADMA + DMA)/SDMA (Figure 1). In 24 h-collected urine samples, the amounts excreted within a day can be used in these terms. In urine samples collected by spontaneous micturition, the creatinine-corrected concentrations of ADMA, DMA, and SDMA are considered.

MMA, SDMA, ADMA, and DMA are natural compounds, and the ingestion of vegetable and meaty food may contribute to endogenously produced SDMA, ADMA, and most notably to DMA [25,26,27]. Application of the above described protein Arg dimethylation concept in health and disease requires taking proper measures to minimize exogenous contributors to urinary SDMA, ADMA, and DMA. Previously, we found that dietary fat ingestion increased the plasma concentration of ADMA marginally (by 6%) in lean and obese healthy subjects [28]. In 10 overweight men, we previously found that high-fat protein meals (HFMs) acutely increased plasma ADMA concentrations [29]. Yet, in those studies, we did not measure ADMA, DMA, and SDMA in urine samples.

The aim of the present study was to apply the above proposed indices of Arg methylation to a human study, and to test the hypothesis that dimethylarginine methylation in proteins would increase after a HFM. We measured, by fully validated and previously reported gas chromatography–mass spectrometry (GC–MS) methods, the concentration of ADMA, DMA, SDMA, and creatinine in spot urine samples collected in previous study [30] before, during, and after HFM meals consumed on three occasions by the 10 healthy overweight volunteers.

## 2. Materials and Methods

### 2.1. Ingestion of High-Fat Protein Meals by Healthy Overweight Men

The urine samples analyzed in the present study had been collected in a previous study, reported by us in detail [30]. The study was conducted in accordance with the Declaration of Helsinki, approved by the Ethics Committee of Saint-Germain-en-Laye Hospital (Reference #08001), and authorized by the French Ministry for Health (Reference 2007-A01296-47). All participants gave their written informed consent prior to enrolment. The study recruited eleven healthy overweight (body max index (BMI)> 25 kg/m²) men aged 21–50 years, with enlarged waist circumference (>94 cm), and without any established illnesses. This sample size was set taking into account 10% attrition, and considering that 10 individuals were necessary to detect medium effect size (Cohen’s d = 0.5) of the treatment on the primary outcome of the clinical trial (postprandial endothelial dysfunction). Volunteers had no regular use of medication or nutritional supplements, were not heavy smokers or alcohol drinkers, and had no moderate/high level of physical activity. They had blood hemoglobin >130 g/L, and no hypertension. The volunteers had the following characteristics: age, 34 ± 9 years; height, 178 ± 3 cm; weight, 96 ± 6 kg; BMI, 30.2 ± 1.5; body fat, 24.3 ± 2.0%; waist circumference, 96 ± 3 cm. Three HFMs of the same nutritional composition, but differing in the protein source, were tested in a randomized crossover design. Each period consisted of a postprandial study separated by at least two weeks. The test meals consisted of a mixture of 233 g cream containing 40% fat, 45 g sucrose, 45 g protein as protein isolates, and 160 mL water. The composition of the meals was as follows: energy, 1200 kcal; fat, 93 g (70% energy); carbohydrates, 45 g (15% energy); crude protein, 45 g (15% energy). After the overnight fasting (9–12 h), the subjects ingested the meal, and spot urine samples were collected before the meal (0 h, T0) and 2 h (T2), 4 h (T4), and 6 h (T6) after the meal. One subject chose to withdraw from the study during the first session because he felt nauseated after the meal. The urine samples collected during the meal were also analyzed and considered in statistics.

### 2.2. Measurement of Urinary ADMA, DMA, SDMA, Creatinine, and Quality Control

Creatinine, ADMA, DMA, and SDMA were measured by previously reported fully validated methods based on gas chromatography–mass spectrometry (GC–MS) methods [22,24,31,32]. Urine donated by a healthy volunteer served as a quality control (QC) sample, and was analyzed alongside the study samples within 8 runs. The following analyte concentrations were measured in the QC samples (mean ± SD): 10.7 ± 0.02 mmol/L (RSD, 2%) for creatinine, 21.3 ± 0.4 µmol/L (RSD, 2.0%) for ADMA, 36.2 ± 1.27 µmol/L (RSD, 2%) for SDMA, and 243 ± 18 µmol/L (RSD, 7.4%) for DMA. These results underline the reliability of the GC–MS methods in the measurements of the study samples.

### 2.3. Measurement of Inflammation and Cardiovascular Biomarkers

The biochemical parameters apolipoprotein B 48 (apoB48), monocytes chemoattractant protein-1 (MCP-1), myeloperoxidase (MPO), non-esterified fatty acids (NEFA), reflexion index (RI), soluble intracellular adhesion molecule-1 (sICAM-1), soluble vascular cell adhesion molecule-1 (sVCAM-1), triacylglyceride (TAG), tumor necrosis factor-alpha (TNF-α), tissue plasminogen activator inhibitor-1 (tPAI-1), and the physiological parameters reflexion index (RI) from pulse wave analysis were measured as described elsewhere [30].

### 2.4. Statistical Analyses

Statistical analyses were performed, and graphs were constructed using Origin 7.5G, GraphPad Prism 7 (GraphPad Prism Software Inc. San Diego, CA, USA). Distribution of variables was tested by D’Agostino and Pearson omnibus K2 test. Normally distributed parameters are presented as mean ± SD or mean ± SEM. Non-normally distributed parameters are presented as median and interquartile range (25th–75th percentile). Correlations between variables were assessed by Pearson (parametric) or Spearman (non-parametric) statistical tests. Repeated measures one-way ANOVA with Tukey’s multiple comparisons test was used to test the effect of postprandial time on the urinary parameters. *p*-values < 0.05 were considered as statistically significant.

## 3. Results

Considering all volunteers, meals, and time points (*n* = 124 values), the urinary analyte concentrations were 40.2 (22–57) µmol/L for ADMA, 55.2 (31–76) µmol/L for SDMA, 348 (180–536) µmol/L for DMA, and 11.9 (6.7–19.6) mmol/L for creatinine. The creatinine-corrected excretion rates (µmol/mmol) were 3.39 (2.50–4.15) for ADMA, 4.48 (3.33–5.78) for SDMA, and 29 (21.7–37.0) for DMA. The other values were 32.8 (24.6–41.1) µmol/mmol for aPADiMeX, 37.9 (28.5–47.0) µmol/mmol for toPADiMeX, and 7.26 (6.27–8.20) for a/sPADiMeX. The concentrations of ADMA, SDMA, DMA, and creatinine correlated strongly with each other (Table 1a). The creatinine-corrected concentrations of ADMA, SDMA, and DMA also correlated with each other (Table 1b).

At baseline, many of the plasma clinical chemistry biochemical parameters correlated moderately-to-strongly with each other (Table 2). All of the found statistically significant correlations were positive, except for NEFA and tPAI-1. The strongest correlation was observed between IL-6 and TNF-α (*r* = 0.847, *p* < 0.0001).

The correlations found between plasma clinical chemistry biochemical parameters and the creatinine-uncorrected urinary concentrations of ADMA, SDMA, DMA, and toPADiMeX at baseline are summarized in Table 3. These parameters correlated with TNF-α and IL-6, with SDMA showing the strongest correlation. The BMI value of the volunteers (range 26.9–33.4 kg/m^2^) was found to correlate with insulin, sICAM-1, sVCAM-1, and E-selectin (Table 3). At baseline, the urinary concentrations (µmol/L) of ADMA, SDMA, DMA, and toPADiMeX, or with a/sPADiMeX, did not correlate with the BMI values. The creatinine-corrected excretion rates of DMA (*r* = −0.354, *p* = 0.051) and toPADiMeX (*r* = −0.324, *p* = 0.076) only tended to correlate with the BMI (not shown in Table 3).

We did not observe any postprandial changes in the indices for the individual HFM (data not shown), and so the dataset of the 3 individual meals (*n* = 10) were collapsed to a single dataset (*n* = 30). The urinary creatinine concentration, the creatinine-corrected excretion of ADMA, DMA, and SDMA, and their indices aPADiMeX, toPADiMeX, and a/sPADiMeX are summarized in Table 4 for all three meals. Statistically significant overall time effects (ANOVA) were obtained for creatinine (*p* = 0.0021) and DMA (*p* = 0.019). Statistically significant time effects were obtained for creatinine (T0 vs. T4, *p* = 0.0007; T4 vs. T6 *p* = 0.0011), ADMA (T2 vs. T6, *p* = 0.0006), DMA (T0 vs. T4, *p* = 0.0027), SDMA (T4 vs. T6, *p* = 0.0499), and aPADiMeX (T0 vs. T4, *p* = 0.0290).

## 4. Discussion

In blood and urine, MMA is present at much lower concentrations than ADMA and SDMA. For instance, in healthy humans, the mean creatinine-corrected excretion rate of MMA was reported to be 0.017 µmol/mmol, and the ratio of the mean clearance rates of MMA, ADMA, and SDMA were reported to be 1:69:71 [33]. This may be an indicator that MMA, the first PRMT-catalyzed product of Arg-methylation in proteins, is immediately methylated to form ADMA and SDMA proteins. Other ADMA and SDMA metabolites from *N^α^*-acetylation and *N^α^*-oxidation pathways occur in urine, yet at much lower concentrations than ADMA and SDMA, such as 0.013 µmol/mmol creatinine, and in the range 0.011–1.03 µmol/mmol creatinine for the ADMA metabolites, respectively [34,35]. Consequently, the urinary concentrations of ADMA, DMA, and SDMA are useful for the determination of the whole-body *N*^G^-dimethylation of Arg residues in proteins.

In the present work, we propose the use of the Protein Arginine Dimethylation indeX: aPADiMeX for asymmetric, sPADiMeX for symmetric, toPADiMeX for total dimethylation, and a/sPADiMeX for the asymmetric-to-symmetric (a/s) dimethylation state (Figure 1). We applied this proposal to (1) investigate potential postprandial effects of HFMs on protein Arg dimethylation; and (2) to test potential correlations of the indices with clinical chemistry laboratory biomarkers of inflammation and vascular functions, such as IL-6 and TNF-α.

The ADMA/SDMA molar ratio in our study is close to 1, and is almost identical with that reported for healthy subjects [33]. However, this ratio does not mean that asymmetric and symmetric protein Arg dimethylation rates are equal. This is because ADMA is metabolized to DMA, of which the excretion is about 10 times higher than non-metabolized ADMA in healthy adults [24,33].

The creatinine-corrected excretion rates of the ADMA, DMA, and SDMA measured in the present study at baseline, are within ranges reported by us and others for healthy and diseased adults [24,33]. In our healthy overweight men, the average baseline creatinine-corrected urinary excretion rates (µmol/mmol) were 3.59 for ADMA, 4.48 for SDMA, and 26.9 for DMA. The baseline indices were calculated to be 30 µmol/mmol for aPADiMeX, 37.1 µmol/mmol for toPADiMeX, and 6.7 for a/sPADiMeX. These data indicate that DMA is the strongest quantitative contributor to the proposed indices. The whole-body asymmetric dimethylation of proteinic Arg is about 7 times higher than the symmetric in the healthy overweight men of the present study.

In urine samples from 14 healthy non-overweight men (age, 41 ± 11 years; range, 26–60 years; BMI, 23.9 ± 3.3 kg/m^2^) from previous work [32], we measured creatinine-corrected excretion rates of 29.6 ± 3.9 µmol/mmol DMA, 2.74 ± 0.51 µmol/mmol ADMA, and 2.99 ± 0.44 µmol/mmol SDMA. The Arg dimethylation indices were calculated to be 35.0 ± 5.2 µmol/mmol for aPADiMeX, 38.0 ± 5.5 µmol/mmol for toPADiMeX, and 11.8 ± 1.6 for a/sPADiMeX. In urine samples from 5 healthy non-overweight women (age, 42 ± 10 years; range, 28–56 years; BMI, 26.9 ± 7.8 kg/m^2^) from the same study [32], we measured creatinine-corrected excretion rates of 42.5 ± 5.8 µmol/mmol DMA, 4.2 ± 1.2 µmol/mmol ADMA, and 3.91 ± 0.6 µmol/mmol SDMA. The Arg dimethylation indices were calculated to be 46.7 ± 6.6 µmol/mmol for aPADiMeX, 50.6 ± 7.2 µmol/mmol for toPADiMeX, and 10.8 ± 0.8 for a/sPADiMeX. Statistically significant differences between men and women were found for DMA (*p* = 0.01), ADMA (*p* = 0.003), SDMA (*p* = 0.022), aPADiMeX (*p* = 0.007), and toPADiMeX (*p* = 0.0046), but not for a/sPADiMeX (*p* = 0.95), suggesting potential effects of gender on whole-body asymmetric and symmetric proteinic Arg dimethylation, yet not on their balance.

In previous studies, we found that HFM taken by the same healthy overweight men is associated with considerable postprandial changes in many circulating biochemical biomarkers, including Arg, L-homoarginine (hArg), and ADMA [29,30,36]. The present study indicates that HFM has no appreciable postprandial effects on total asymmetric and symmetric protein arginine dimethylation (toPADiMeX), and asymmetric-to-symmetric protein arginine dimethylation (a/sPADiMeX). However, we found some significant temporary changes on ADMA (decrease, between T2 and T6), DMA, SDMA, and aPADiMeX (increases, all between T0 and T4). At baseline, circulating TNF-α and IL-6 correlated with urinary creatinine-corrected SDMA excretion. Previously, we found no statistically significant changes in circulating TNF-α and IL-6 upon meal ingestion [30], suggesting that these factors may not be responsible for the observed changes in SDMA excretion. The results of the present study may suggest that the HFM themselves did not contain appreciable amounts of ADMA, DMA, and SDMA (not investigated), and did not exert appreciable effects on dimethylation of proteinic Arg. As creatinine excretion changed relatively strongly, an effect on the glomerular filtration rate (GFR) of the kidney on the excretion rates of ADMA, DMA, and SDMA cannot be excluded. In renal transplant recipients (median estimated GFR of 43.5 mL/min/1.73 m^2^), the mean ADMA-to-SDMA molar ratio was found to be only 0.6 [37].

The non-invasive measurement of ADMA, DMA, and SDMA in human urine provides a relevant approach to estimate the extent of proteinic Arg dimethylation and the relative contribution of the asymmetric and symmetric dimethylation, which can be translated into the individual PRMTs. The proposed indices may provide valuable information of the status of protein arginine dimethylation in health and disease. In healthy overweight men, HFM ingestion caused temporary changes in creatinine excretion, and creatinine-corrected excretion rates of ADMA, DMA, and SDMA. However, these changes do not indicate changes in the total protein arginine dimethylation, and the balance between asymmetric and symmetric protein arginine dimethylation. Of note, asymmetric dimethylation tended to increase after HFMs.

A limitation of our study is the small number of participants. Strengths of the study are the closely controlled, repeated postprandial testing on the same volunteers, controlled meal composition, and validated methods for measuring arginine metabolites in urine. Urinary DMA is by far the greatest term of the indices proposed in this work. Further studies on larger cohorts are warranted to assess potential differences in methylation profiles of proteinic arginine in various conditions in health and disease, and to assess the effects of gender and age. In studies addressing the in vivo protein arginine dimethylation in non-closely controlled studies, subjects must abstain from ingestion of DMA-rich food, notably fish [24,25,26,27].

## Figures and Tables

**Table 1:** Spearman correlation coefficients between the concentrations (**a**) and creatinine-corrected excretion rates (**b**) of the analytes in the urine samples for all volunteers, meals, and time points (*n* = 124 values). toPADiMeX is ADMA + DMA + SDMA for total Arg dimethylation.

**Table 1a**	**ADMA**	**SDMA**	**DMA**
SDMA (µmol/L)	0.939, *p* < 0.0001		
DMA (µmol/L)	0.900, *p* < 0.0001	0.923, *p* < 0.0001	
Creatinine (mmol/L)	0.842, *p* < 0.0001	0.886, *p* < 0.0001	0.883, *p* < 0.0001
**Table 1b**	**ADMA**	**SDMA**	**DMA**
SDMA (µmol/mmol)	0.811, *p* < 0.0001		
DMA (µmol/mmol)	0.652, *p* < 0.0001	0.765, *p* < 0.0001	
toPADiMeX (µmol/mmol)	0.755, *p* < 0.0001	0.842, *p* < 0.0001	0.981, *p* < 0.0001

**Table 2:** Spearman correlation coefficients between plasma clinical laboratory parameters at baseline.

	Glucose	TAG	apoB48	NEFA	Insulin	TNF-α	IL-6	MCP-1	sICAM-1	sVCAM-1	MPO	E-Selectin	tPAI-1	RI
TAG	0.086													
apoB48	0.034	0.785 ^c^												
NEFA	−0.265	0.099	0.055											
Insulin	0.249	−0.225	−0.062	−0.312										
TNF-α	0.395 ^a^	−0.082	−0.010	−0.154	0.372 ^a^									
IL-6	0.489 ^a^	−0.026	0.023	−0.158	0.446 ^a^	0.847 ^c^								
MCP-1	0.109	−0.116	0.077	−0.301	0.557 ^b^	0.690 ^c^	0.754 ^c^							
sICAM-1	−0.235	0.076	0.121	−0.218	0.495 ^a^	0.198	0.164	0.283						
sVCAM-1	−0.231	−0.048	0.090	−0.471 ^a^	0.303	−0.012	−0.059	0.131	0.726 ^c^					
MPO	−0.187	−0.050	−0.108	−0.164	0.073	−0.212	−0.108	0.118	−0.010	0.063				
E-Selectin	−0.242	−0.281	−0.224	−0.403 ^a^	0.454 ^a^	0.216	−0.028	0.225	0.630 ^c^	0.598 ^c^	0.203			
tPAI-1	0.126	−0.171	−0.153	−0.061	0.089	0.004	0.168	0.016	0.059	−0.073	0.039	0.168		
RI	−0.104	−0.163	−0.169	−0.467 ^a^	0.197	0.055	−0.074	0.033	0.348	0.485 ^a^	−0.092	0.565 ^b^	0.111	
SI	−0.087	0.436 ^a^	0.317	0.296	−0.022	0.017	0.058	0.118	−0.025	−0.156	0.136	−0.226	−0.673	−0.236

^a^, *p* < 0.05; ^b^, *p* < 0.001; ^c^, *p* < 0.0001.

**Table 3:** Spearman correlation coefficients between baseline urinary concentrations (µmol/L; not corrected for creatinine) of ADMA, SDMA, DMA, toPADiMeX, and a/sPADiMeX, BMI, and plasma clinical chemistry biochemical parameters. Data are not shown for correlations among the plasma parameters (i.e., from Glucose to Basal_SI). (*), 0.05 < *p* < 0.1; *, *p* < 0.05; **, *p* < 0.001; ***, *p* < 0.0001.

	ADMA	SDMA	DMA	toPADiMeX	a/sPADiMeX	BMI
BMI	0.072	0.168	0.057	0.064	−0.279	
Glucose	0.249	0.328 (*)	0.312 (*)	0.332 (*)	−0.068	−0.191
TAG	−0.143	−0.141	−0.164	−0.180	−0.125	−0.235
apoB48	−0.285	−0.260	−0.236	−0.264	−0.017	−0.076
NEFA	0.171	0.167	0.156	0.150	−0.107	−0.117
Insulin	0.197	0.240	0.160	0.189	−0.205	0.458 *
TNF-α	0.360 *	0.526 **	0.392 *	0.422 *	−0.241	0.310 (*)
IL-6	0.349 (*)	0.496 **	0.370 *	0.411	−0.298	0.200
MCP-1	0.116	0.279	0.172	0.198	−0.212	0.339 (*)
sICAM-1	0.134	0.126	−0.021	−0.013	−0.376 *	0.627 ***
sVCAM-1	−0.207	−0.208	−0.291	−0.302	−0.208	0.415 *
MPO	−0.214	−0.187	−0.217	−0.223	−0.233	0.113
E-Selectin	−0.103	−0.034	−0.150	−0.147	−0.257	0.774 ***
tPAI-1	−0.001	0.004	0.080	0.071	0.094	0.295
Basal_RI	0.066	−0.012	0.056	0.053	0.186	0.217
Basal_SI	0.005	0.008	−0.126	−0.103	−0.317 (*)	−0.211

**Table 4:** Urinary creatinine (mmol/L), creatinine-corrected excretion of ADMA, DMA, SDMA, aPADiMeX, toPADiMeX (µmol/mmol), and a/sPADiMeX (median (25th–75th) or mean ± SD) at the indicated time points.

Measure	T0	T2	T4	T6	ANOVA
**Creatinine**	10.9 (5.5–17.0)	12.0 (8.6–21.9)	17.4 ± 9.5 T4 vs. T0: *p* = 0.0007	8.9 (5.2–15.4) T6 vs. T4: *p* = 0.0011	*p* = 0.0021
**ADMA**	3.59 ± 1.38	3.62 ± 1.3	3.43 ± 1.23	3.17 ± 1.09 T6 vs. T2: *p* = 0.0006	*p* = 0.0718
**DMA**	26.9 (19.9–35.1)	27.5 (20.3–36.1)	33.7 (22.2–37.3) T4 vs. T0: *p* = 0.0027	30.8 ± 10.1	*p* = 0.0190
**SDMA**	4.45 (3.48–5.59)	4.78 (3.1–6.3)	4.52 (3.35–5.86)	4.05 (3.22–5.64) T6 vs. T2: *p* = 0.0499	*p* = 0.2687
**aPADiMeX**	29.9 (24.3–39)	30.7 (22.2–40.4)	37.4 (24.8–41.9) T4 vs. T0: *p* = 0.0290	33.9 ± 10.9	*p* = 0.0677
**toPADiMeX**	33.5 (29.9–44.0)	33.8 (28.2–45.4)	37.6 (26.1–43.0)	38.3 ± 11.4	*p* = 0.2980
**a/sPADiMeX**	7.21 (5.25–8.63)	7.57 ± 3.15	7.90 ± 2.81	8.46 ± 3.19	*p* = 0.2213
